# On-line identification of the end of motor imageries based on the alpha rebound detection

**DOI:** 10.1186/1471-2202-16-S1-P207

**Published:** 2015-12-18

**Authors:** Cecilia Lindig-León, Laurent Bougrain, Sébastien Rimbert

**Affiliations:** 1 Inria, Villers- lès -Nancy, F-54600, France; 2 Université de Lorraine, LORIA, UMR 7503, Vandœuvre-lès-Nancy, F-54500, France; 3 CNRS, LORIA, UMR 7503, Vandœuvre-lès-Nancy, F-54500, France

## 

Limb movement execution and imagination elicit in a mutually exclusive manner sensorimotor rhythms that can be detected in electroencephalographic (EEG) recordings; in particular over the primary motor cortex, where an oscillatory modulation has been observed prior, during and following the execution of voluntary movement, passive movement, imagined movement, and even tactile stimulation [1,2]. The modulation following the movement termination consists of an event-related synchronization (ERS) that increases the oscillatory power for a few hundred milliseconds [3]. Since it is known to be specific of the beta band 13-25 Hz, it is denoted as post-movement beta rebound, although in recent studies it has been shown that this phenomenon is enhanced when analyzed in the alpha range 8-13 Hz (see Figure 1). [1,4].

**Figure 1:**
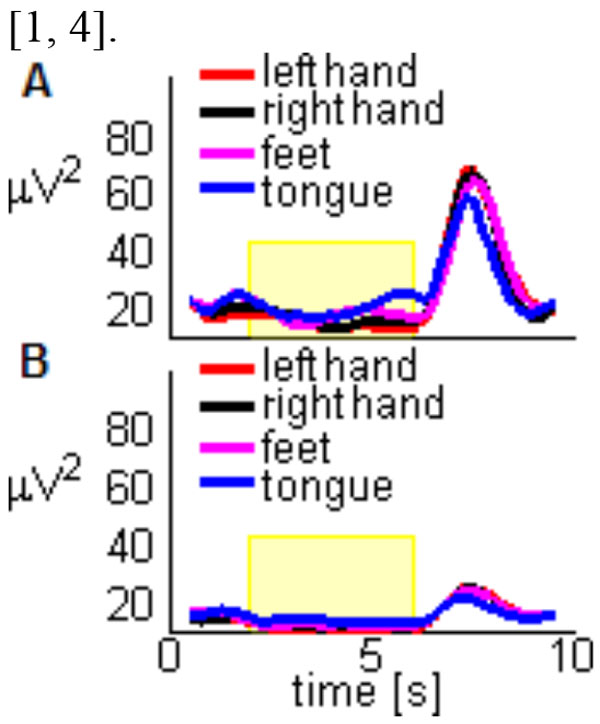
**Averaged post-movement rebound for subject 7 in electrode C4 across the A**. alpha and B. beta bands. The yellow box indicates the duration of the motor imageries.

The characteristics of this post-movement rebound, as it will be shown in the present study, are preserved independently of the involved limb during the motor execution. From database 2a of the BCI competition IV [4], an on-line method for identifying the end of motor imageries on a single trial detection is presented. By using an overlapped sliding window over each trial from four different motor imageries (left hand, right hand, feet and tongue), two contrasting classes are generated according to the occurring condition (i.e., segments with rebound and segments without it) to generate a classification model based on a linear discriminant analysis. Results show that the classification performance is 5% superior over the alpha band than the beta band for almost all subjects, and that the rebound detection is independent from the limb used in the motor imagery.

## Conclusions

On-line detection of the end of motor imageries of various body parts is feasible by detecting the post-movement alpha rebound. The accuracy reached by the proposed method within the alpha band across all subjects is 79.17% with a sensitivity value of 0.81 and specificity of 0.71. This method improves the detection of the end of motor imageries by considering the alpha post-movement rebound, which is of interest for the design of self-paced brain-computer interfaces.
